# Development and evaluation of a rehabilitation training compliance scale for patients with urinary incontinence

**DOI:** 10.1186/s12912-023-01326-5

**Published:** 2023-05-04

**Authors:** Liumei Luo, Xi Chen, Huifang Xie, Jiaquan Zhou, Li Li

**Affiliations:** 1 Teaching and Research Section of Clinical Nursing, Xiangya Hospital of Central South University, Changsha, 410008 China; 2 Department of Scientific Research, Hainan General Hospital, Haikou, 570311 China; 3 National Clinical Research Center for Geriatric Diseases, Xiangya Hospital, Central South University, Changsha, 410008 China

**Keywords:** Urinary incontinence, Rehabilitation training, Compliance, Scale, Evaluation

## Abstract

**Background:**

Urinary incontinence treatment includes conservative treatment, physical devices, medication, and surgery. Pelvic floor muscle training combined with bladder training is among the most effective, non-invasive, and economical ways to treat urinary incontinence, and compliance with training is essential in urinary incontinence treatment. Several instruments assess pelvic floor muscle training and bladder training. However, no tool has been found that assesses compliance with pelvic floor muscle training when combined with bladder training for urinary incontinence. This study aimed to develop a rehabilitation training compliance scale for patients with urinary incontinence and to evaluate its validity and reliability.

**Methods:**

This study was performed in two tertiary hospitals in Hainan, China between December 2020 and July 2021, 123 patients were included. A literature review, group discussions, and two rounds of letter consultations were performed to acquire the item pool and finalise the 12 items for this scale. Exploratory and confirmatory factor analysis, Cronbach’s α, split-half reliability, test–retest reliability, content validity, construct validity, convergent and discriminant validity, and criterion-related validity were used to examine the items in the scale.

**Results:**

A 12-item scale comprising three factors accounted for 85.99% of the variance in the data. The Cronbach’s α, split-half reliability, test–retest reliability, and content validity index of the scale were 0.95, 0.89, 0.86, and 0.93, respectively. Comparison with the Chen pelvic floor muscle exercise self-efficacy scale showed high calibration correlation validity (coefficient = 0.89).

**Conclusions:**

The training compliance scale developed in this study is a valid and reliable measurement tool to assess pelvic floor muscle training and bladder training compliance in patients with urinary incontinence.

## Background

Urinary incontinence (UI) refers to an objectively proven condition of involuntary urine leakage [1]. Sneezing and coughing can induce urine leakage, which leads to awkwardness, anxiety, and depression in most patients. The frequent urine leakage and the consequent unpleasant smell may deprive patients of socialisation and even cause sleeping disorders in some patients, which consequently induces psychological diseases [2]. UI has a high incidence and affects a wide range of people, influencing the health of patients and the lives of their families. Studies in the United States, the United Kingdom, and Sweden have shown that the incidence of UI is as high as 46% and 68% in males and females, respectively [3]. The incidence of UI was 8.7% to 69.8% in Chinese women, representing 43–349 million women [4]. UI can cause a number of sexual dysfunctions, and 83.45% of patients were dissatisfied with their sex lives [5]. Patients with UI also experience decreased quality of life [6]. UI not only has an adverse influence on patients and their families but also increases the disease burden on society [7].

UI treatment includes conservative treatment (e.g. appropriate fluid intake, weight loss, smoking cessation, and rehabilitation training), physical devices, medication, and surgery [8, 9]. Compared with surgical treatments, which are associated with substantial trauma and high costs, conservative treatments are effective, safe, and acceptable and have been considered as the major treatment for UI [9]. The International Urinary Control Association recommends pelvic floor muscle and bladder training as the first-line treatment for patients with UI, affirming the role of such training in improving UI [10]. Studies have demonstrated that pelvic floor muscle and bladder exercises are effective training methods. Most evidence has shown that pelvic floor muscle training combined with bladder training is more effective than pelvic floor muscle training alone [8, 11]. Pelvic floor muscle training (PFMT) refers to patients consciously training their pelvic floor muscles, mainly the pubic coccygeus muscle group, to autonomously contract [12, 13]. Bladder training (BT) refers to patients urinating at prescribed times and gradually lengthening the intervals between urination to gradually increase their bladder capacity and enhance their control of the bladder function [14, 15]. These exercises are simple and easy to perform and suitable for patients capable of autonomous training [16, 17], but compliance is often problematic [18]. Compliance with pelvic floor muscle and bladder training plays an important role in improving pelvic floor muscle and bladder function and has been proven a main predictor of exercises long-term effectiveness [17]. Compliance is the extent to which a patient’s behaviour complies with the doctor’s advice regarding the treatment and prevention of disease [19]. In this study, the concept of compliance is defined as the degree of patients’ compliance with doctors’, therapists’, and nurses’ advice, consisting of the degree of consistency in the frequency, duration, and initiative of pelvic floor muscle training and bladder training. Training compliance is influenced by multiple factors and requires patients’ active participation [18]. Poor training compliance, for example in patients who forget to complete training, can lead to minimal perceived benefits [18, 20]. Therefore, it is crucial to develop a training compliance scale for patients with UI. This scale can assess the training compliance in patients with UI, which can help doctors to predict the efficacy of training and patients’ recovery. If the scale assessment outcome shows that a patient’s training compliance is poor, more strategies should be used to help the patient’s recovery.

To systematically published literature search data, there is a large body of research regarding pelvic floor muscle training [21] and bladder training [8]. However, most of these studies have focused on the mechanisms [22], treatment methods [23], and treatment effects [24] of pelvic floor muscle training for UI in women. Some studies have focused on the treatment effects of PFMT combined with BT for UI in women [11]. Few studies have reported on PFMT and BT compliance [18, 25], and we did not find any studies that developed a training compliance scale, although several studies have used a pelvic floor muscle exercise self-efficacy scale for female patients, like the Chen’s pelvic floor muscle exercise self-efficacy scale [26–28]. To the best of our knowledge, no study has developed a compliance scale for PFMT combined with BT for patients with UI. Therefore, this study aimed to develop a training compliance scale for patients with UI and evaluate its validity and reliability. Our findings may provide guidance regarding the assessment of training compliance of UI patients, which would help to increase their compliance with pelvic floor muscle and bladder training, improve their quality of life, and promote their recovery.

## Methods

### Study design and participants

This study developed and evaluated a training compliance scale for patients with UI in three steps: 1) establishment of the item pool and development of the scale; 2) evaluation of the validity of items, as well as the validity and reliability of the scale; and 3) exploratory factor analysis and confirmatory factor analysis.

There were 10 participants in the group discussions: one urological surgeon and one gynaecologist with senior professional titles, one urological surgeon and one gynaecologist with medium-grade professional titles, two nurses with senior professional titles, two nurses with medium-grade professional titles, and two nurses with master’s degrees in nursing science. All the inclusion members were included following the inclusion criteria: 1) Doctors or nurses engaged in urology or gynecology; 2) Bachelor's degree with at least 5 years of work experience, or master's degree and familiar with the field; 3) Volunteer to participate in the study.

A team of 22 experts participated in a Delphi survey. The inclusion criteria for these experts were as follows: 1) having at least a bachelor’s degree; 2) having at least 10 years’ work experience; 3) having a job level of associate senior or above; 4) being a physician, rehabilitation therapist, or nurse engaged in the diagnosis and treatment of UI or UI rehabilitation; and 5) being willing to participate in the consultation and able to complete the consultation on time. The team members were two chief nurses in charge of the daily management of nursing, eight chief doctors in charge of the management of medical practices, and 12 experts from UI-related departments (urology, obstetrics and gynaecology, and rehabilitation departments).

Convenience sampling was used to enrol patients diagnosed with UI who were referred to the outpatient departments of two tertiary 3A hospitals in Hainan, China for re-examinations between December 2020 and July 2021. The inclusion criteria for these patients were as follows: 1) a diagnosis of UI, overflow incontinence, UI following prostatectomy, or UI following in situ ileal neobladder [29–31], and the presence of UI as the prominent disease; 2) an age of at least 18 years; 3) a clear conscience and the ability to express themselves; 4) willingness to volunteer for the study; and 5) having received guidance on recovery training. Participants who had participated in similar studies before were excluded from the study.

### Scale development

First, a literature analysis was performed to develop a training compliance scale for patients with UI. The Web of Science, PubMed, CNKI, and WANFANG databases were searched for relevant literature before October 25, 2020. The search terms were as follows: (‘urinary incontinence’) AND (‘pelvic floor muscle training (PFMT)’ OR ‘bladder training’ OR ‘bladder exercise’) AND (‘compliance’ OR ‘adherence’) AND (‘scale’ OR ‘gauge’ OR ‘questionnaire’ OR ‘questionnaire survey’ OR ‘compliance scale’ OR ‘compliance gauge’). The corresponding Chinese terms were used as the search terms in the Chinese-language databases (CNKI and WANFANG).

A priori eligibility criteria identified in the protocol were used to identify studies for inclusion. These criteria were as follows: (1) being conducted among adults aged at least 18 years with a diagnosis of UI; and (2) being related to rehabilitation training compliance in patients with UI. After a comprehensive review of the relevant literature on pelvic floor muscle training [2, 26, 32], bladder training [33–37], and voiding diaries [38–40], 25 candidate items were identified for the scale. Please see Table 1.

**Table 1:** Items in the item pool and their sources

Items	Source
1. Do you contract your pelvic floor muscles?	[12, 58]
2. I can continue to contract my pelvic floor muscles for 3 s or more each time, relax, and rest for 2 to 6 s	[58, 59]
3. I can repeat the above (last) action for 15 to 30 min	[12, 58]
4. I can do pelvic floor muscle training three times a day	[26]
5. I can continue to contract my pelvic floor muscles for 2 to 6 s each time, relax, and rest for 2 to 6 s	[61]
6. I can repeat the above action (the previous one) 10 to 15 times	[34, 60, 61]
7. I can do the above actions (the last two) and train 3 to 8 times a day	[12, 61]
8. I can do pelvic floor muscle training at any time and in any place	[26]
9. I can do pelvic floor muscle training when standing, sitting, and lying down	[62]
10. I can persist in pelvic floor muscle training for 8 weeks	[26, 58, 59]
11. I can persist in pelvic floor muscle training for 2 to 3 months	[61, 63]
12. I can do bladder training	[34, 61, 64]
13. I can do bladder training every day	[64]
14. Before going to the toilet each time, I can contract my pelvic floor muscles until the sense of urgency disappears and then relax, and then urinate after 1 to 3 min	[34, 64]
15. I can prolong the urination time	[61]
16. I can control the urination time and gradually extend it to once every 2–3 h	[34, 61]
17. I can gradually prolong the interval between urination and urinate once every 2–3 h	[64]
18. I can delay urination and make the volume of urination more than 300 ml each time	[61]
19. I can delay urination so that the urination volume is less than 350 ml each time	[64]
20. I can urinate regularly	[61]
21. I can urinate regularly once every 2 h in the daytime and once every 4 h at night	[37, 61, 62, 64]
22. I can persist in bladder training for more than 4 to 8 weeks	[61, 62, 64]
23. I can record a urination diary	[34, 39, 61]
24. I can record a urination diary every day	[62, 64]
25. I can record a 24-h urination diary for more than 1 week	[62, 64]

Second, group discussions were held to modify the scale items. The group discussions were held three times, once a week, with a duration of an hour each. A round-table format was used, and the meeting was conducted in the urology conference room, chaired by a physician with a senior title from the discussion group. After the group discussions, 13 items were deleted or combined because of their similarity or redundancy to other items, and modifications were made to ensure that terms were used accurately and that the items were easy to understand. The scale consisted of 12 items after the group discussions. Please see Table 2.

**Table 2:** Changes to the scale based on the group discussions

Old item before group discussion	New item after group discussion	Delete, combine, modify, or keep
1. Do you contract your pelvic floor muscles?	1. I can continuously contract my pelvic floor muscles for 2 to 6 s, relax, and rest for 2 to 6 s	Combined the old items 1 and 5 to become the new item 1
2. I can continue to contract my pelvic floor muscles for 3 s or more each time, relax, and rest for 2 to 6 s		Deleted the old item 2 because it was redundant
3. I can repeat the above (last) action for 15 to 30 min		Deleted the old item 3 because it was redundant
4. I can do pelvic floor muscle training three times a day		Deleted the old item 4 because it was redundant
5. I can continue to contract my pelvic floor muscles for 2 to 6 s each time, relax, and rest for 2 to 6 s		Combined the old items 1 and 5 to become the new item 1
6. I can repeat the above action (the previous one) 10 to 15 times	2. I can repeat the above action (item 1) 10 to 15 times	Kept the old item 6 as the new item 2
7. I can do the above actions (the last two) and train 3 to 8 times a day	3. I can do the above actions (items 1 and 2) and train 3 to 8 times a day	Kept the old item 7 as the new item 3
8. I can do pelvic floor muscle training at any time and in any place		Deleted the old item 8 because it was redundant
9. I can do pelvic floor muscle training when standing, sitting, and lying down	4. I can do pelvic floor exercises when standing, sitting, and lying down	Modified the old item 9 as the new item 4
10. I can persist in pelvic floor muscle training for 8 weeks	5. I can persist in pelvic floor muscle training for 8 weeks	Kept the old item 10 as the new item 5
11. I can persist in pelvic floor muscle training for 2 to 3 months		Deleted the old item 11 because it was redundant
12. I can do bladder training		Combined the old items 12, 13, and 22 to become the new item 10
13. I can do bladder training every day		Combined the old items 12, 13, and 22 to become the new item 10
14. Before going to the toilet each time, I can contract my pelvic floor muscles until the sense of urgency disappears and then relax, and then urinate after 1 to 3 min	6. Before going to the toilet every time, I can contract my pelvic floor muscles until the sense of urgency disappears and then relax, and then urinate after 1 to 3 min	Kept the old item 14 as the new item 6
15. I can prolong the urination time		Deleted the old item 15 because it was redundant
16. I can control the urination time and gradually extend it to once every 2 to 3 h	7. I can gradually extend the interval between two urinations to 2 to 4 h	Combined and modified the old items 16 and 17 to become the new item 7
17. I can gradually prolong the interval between urination, and urinate once every 2 to 3 h		Combined and modified the old items 16 and 17 to become the new item 7
18. I can delay urination and make the volume of urination more than 300 ml each time		Deleted the old item 18 because it was redundant
19. I can delay urination so that the urination volume is less than 350 ml each time	8. I can delay urination so that the urination volume is less than 350 ml each time	Kept the old item 19 as the new item 8
20. I can urinate regularly		Deleted the old item 20 because it was redundant
21. I can urinate regularly once every 2 h in the daytime and once every 4 h at night	9. I can urinate regularly once every 2 h in the daytime and once every 4 h at night	Kept the old item 21 as the new item 9
22. I can persist in bladder training for more than 4 to 8 weeks	10. I can persist in bladder training for 8 weeks	Combined the old items 12, 13, and 22 to become the new item 10
23. I can record a urination diary		Deleted the old item 23 because it was redundant
24. I can record a urination diary every day	11. I can record a urination diary every day	Kept the old item 21 as the new item 11
25. I can record a 24-h urination diary for more than 1 week	12. I can record a urination diary for 7 days	Modified the old item 25 to the new item 12

Third, Delphi sessions were conducted using paper questionnaires and emails to consult with experts to screen and modify the items. Each round of Delphi took one week, with one week between rounds. This was done to ensure that the scale was concise and understandable and to avoid redundant items. The experts scored the importance and relevance of each item in this scale using a 5-point Likert scale (5 = very important, 4 = important, 3 = fair, 2 = unimportant, and 1 = highly unimportant). The experts could suggest the deletion or detailed modification of an item if they felt that the item was unnecessary or that the description was inaccurate. The experts could also add items or descriptions that had not been included in the scale. Please see Table 3.

**Table 3:** Changes to the scale based on the expert consultations

Item no	First round change	Second round change
1	‘I can continue to contract my pelvic floor muscles for 2 to 6 s, relax, and rest for 2 to 6 s’ was modified to ‘I can continue to contract my pelvic floor muscles for 2 to 10 s, relax, and rest for 2 to 10 s’	
2	‘I can repeat the above actions 10 to 15 consecutive times’ was changed to ‘I can repeat the above actions 10 to 15 consecutive times, or for 5 to 15 min’	
3	No change	
4	No change	
5	‘I can persist in pelvic floor muscle training for 8 weeks’ was revised to ‘I can persist in pelvic floor muscle training for 3 months or more’	
6	No change	
7	No change	‘I can gradually extend the interval between two urinations to 2 to 4 h’ was revised to ‘I can gradually extend the interval between two urinations to 2 to 4 h as far as possible’
8	‘I can delay urination to make each urination less than 350 ml’ was modified to ‘I can delay urination to make each urination more than 300 ml’	
9	No change	
10	‘I can persist in bladder training for 8 weeks’ was revised to ‘I can persist in bladder training for 2 months or more’	
11	‘I can record a urination diary every day’ was changed to ‘I can record a 24-h urination diary’	
12	‘I can record urination diary for 7 days’ was changed to ‘I can record urination diary for 3 to 7 days’	‘I can record urination diary for 3 to 7 days’ was changed to ‘I can record a urination diary for 3 to 7 days’

Our research team members read the references and guidelines and extracted the three dimensions of the scale, namely pelvic floor muscle training, bladder training, and urination diary recording, because pelvic floor muscle training and bladder training methods are simple, easy to carry out, and economical. Items 1 to 5 of the scale were used to assess compliance with pelvic floor muscle training, items 6 to 10 were used to assess compliance with bladder training, and items 11 to 12 were used to assess urinary diary recording.

The investigators in the study consisted of 2 postgraduate nursing students and 5 nursing personnel. They were uniformly trained by an associate chief nurse in a urological surgery department. This training covered the introduction to the scale, measures for obtaining informed consent from patients, dispatching of the questionnaire, and matters requiring attention in completing the scale. The questionnaire consisted of three parts: 1) basic data about the patients, including to sex, age, educational level, type of UI, and frequency of urinary leakage; 2) the training compliance scale for patients with UI developed in this study; and 3) the pelvic floor muscle exercise self-efficacy scale developed by Chen et al. [26]. Electronic or paper questionnaires were dispatched to the patients included in the study after obtaining their consent.

### Reliability and validity

Reliability [41] refers to the consistency and robustness of the results measured by a tool. These results reflect the degree of reliability of the tool (scale). To measure the test–retest reliability of this scale, 30 patients with UI were included in the study for a re-assessment 2 weeks after. After re-assessment, the original questionnaires were recovered for analysis. The reliability of the scale was evaluated by its test–retest reliability and internal consistency reliability [42]. Test–retest reliability was used to assess the scale’s dependability [43]. A score over 0.7 is usually recognised as evidence that the scale is stable. The Cronbach’s α coefficient was used to assess the internal consistency of the scale. In general, a Cronbach’s α of 0.8–0.9 is acceptable and > 0.9 is high [26]. The items in the scale were divided into two equal parts to assess their correlation, with a desired value of split-half reliability over 0.8 [44].

Validity [41] indicates the effectiveness of the tool being evaluated. The content validity index (CVI) was calculated based on the scores assigned to the items by the experts. A CVI of ≥ 0.8 indicates excellent content validity. Each expert was asked to assess the relevance of each item to the corresponding content dimension. A 5-level scoring method was used, where (1 = very unessential, 2 = unessential, 3 = general, 4 = essential, and 5 = very essential) through the Delphi method. Items with a score of 4 or 5 were considered to be relevant to the content being measured [45]. The I-CVI was the ratio of the number of experts who determined the item as relevant (i.e. score ≥ 4) to the total number of experts [45]. The S-CVI was calculated as the average CVI across items. The content validity ratio (CVR) [46] was used to assess whether an item was essential for operating a construct. The CVR was calculated by experts' responses to the following options based on the Likert scale: (1 = very unessential, 2 = unessential, 3 = general, 4 = essential, and 5 = very essential), as the number of experts who determine the item as essential (i.e. score ≥ 4) minus “the total number of specialists∕2”, and this result is divided by “the total number of specialists∕2” [45]. The CVR can range from -1 (perfect disagreement) to 1 (perfect agreement), with a CVR greater than zero meaning that over half of the participants recognised an item as essential [47].

Exploratory factor analysis (EFA) and confirmatory factor analysis (CFA) were used to evaluate the structural validity of the scale. EFA was performed for evaluation, and factorial analysis was performed to evaluate the structural validity of the scale by measuring whether the common factors were in agreement with the structural hypothesis of the scale. In CFA, researchers first raise a hypothesised factor structure and then test it, thereby examining whether the proposed model fits the data. A study mentioned that the sample size of 1:5 (N:q; the number of cases (N) to the number of estimated parameters (q)) qualifies for EFA and CFA [45, 48], but it also pointed out that this is used for minimum recommendations. In this study, the sample size for EFA and CFA was calculated to be 5–10 times as many subjects as the number. Therefore, the required sample content calculation formula is as follows: minimum sample size = 12 Items × 5 times = 60. The total sample was split into two parts, in the EFA and CFA stage according to 12 items: 61 samples for EFA and 62 samples for CFA. AMOS 23.0 (IBM Corp., Armonk, NY, USA) was used to examine whether the factor model constructed from EFA was a good fit for the data. The maximum-likelihood estimation method in AMOS with the covariance matrix generated in PRELIS was used to analyse this model. The chi-square test, relative chi-square (CMIN/DF), root mean square error of approximation (RMSEA), comparative fit index (CFI), normed fit index (NFI), non-normed fit index (TLI), and incremental fit index (IFI) were used to examine the model fit [49].

The average variance extracted (AVE) was used to calculate the scale’s convergent validity. An AVE above 0.50 indicates suitable convergent validity [50]. Discriminant validity can be tested by comparing the square root of a factor’s AVE with the correlation of the specific factor with any of the other factors. When the square root of the AVE is greater than the correlation coefficient, it indicates acceptable discriminant validity [50].

Chen’s pelvic floor muscle exercise self-efficacy (PFMSE) scale and the scale developed in this study were administered to 30 patients, and the results were subjected to correlation analysis. A higher coefficient indicates greater calibration correlation validity. The Cronbach’s α for the Chen PFMSE scale was 0.95, the test–retest reliability was 0.86, and good construct validity was reported [26]. The Chen PFMSE scale has been widely used to assess women’s pelvic floor muscle exercise adherence and confidence [10, 51, 52]. Criterion validity describes the instrument’s correlation with its criteria [53]. Thus, this study chose the Chen PFMSE scale to assess the correlation validity of the training compliance scale for patients with UI.

### Evaluation

The scale developed in this study was scored on a 5-point Likert scale. The total score of the scale ranged from 12 to 60 points. Every item in this scale was assigned a score of 5, 4, 3, 2, or 1 point, indicating ‘always’, ‘usually’, ‘sometimes’, ‘occasionally’, and ‘never’, respectively. The evaluation criteria were as follows: a score ≥ total score of the scale × 80% indicated high compliance, and a score < total score of the scale × 80% indicated poor compliance [54].

### Statistical analysis

SPSS 18.0 (SPSS Inc., Chicago, IL, USA) was used for statistical analysis, and two postgraduate nursing students checked the data independently. If any disagreements arose, the raw data were checked. Categorical data are described using frequencies and percentages, and continuous data are represented using means and standard deviations (SDs). Cronbach’s α was calculated for the dimensions and the scale. The Kappa coefficient and CVI were used for the items in the training compliance scale.

Kappa coefficients can be used to assess the quantifying reliability of scale [55]. The Kaiser–Meyer–Olkin (KMO) value was calculated, and Bartlett’s test of sphericity was conducted. The scale in this study and the pelvic floor muscle training self-efficacy scale developed by Chen were administered to 30 patients at the same time, and correlation analysis was conducted to obtain criterion validity. The larger the calibration validity coefficient, the better the calibration correlation. A two-sided *P* < 0.05 was considered statistically significant.

## Results

### Development of the scale

Three steps were taken to develop the scale. The development result of each step is shown below. During the first step, the literature review was used to build an item pool with 25 items for the scale. The literature review was conducted by a library staff who charge in teaching literature search and the researcher LLM. The details of the items and their sources are shown in Table 1.

During the second step, 10 staff members with rich working experience in a urology department held group discussion three times to modify the items in the scale. After deleting, combining, and modifying the items as needed, 12 items were retained. The details of the modifications made during this step are provided in Table 2.

During the third step, two Delphi rounds were conducted with 22 experts using paper questionnaires and email. No items were deleted during this step, but the experts gave comments to modify some of the items. The details of the modifications made during each round of this step are shown in Table 3.

#### Characteristics of the study participants

In total, 132 questionnaires were dispatched during this study, and 123 validated questionnaires were recovered, yielding an effective recovery rate of 93.18%. These patients consisted of 88 males and 35 females, and their mean age was 58.57 ± 12.96 years (35–83 years). The demographic and clinical characteristics of the 123 participants are shown in Table 4. The score of three dimensions of patients with urinary incontinence compliance scale are shown in Table 5. The items’ score of patients with urinary incontinence rehabilitation training compliance scale are shown in Table 6.

**Table 4:** Demographic and clinical characteristics of the 123 participants

Characteristic	Group	n (%)/SD
Gender	Male	88 (71.5)
	Female	35 (28.5)
Age (year)	/	58.57 ± 12.96
Education	College below	55 (44.7)
	College and above	68 (55.3)
History of pelvic surgery	Yes	122 (99.2)
	No	1 (0.8)
History of pregnancy or birth	Yes	33 (26.8)
	No	90 (73.2)
Types of urinary incontinence	Urine leaks when coughing or laughing	54 (43.9)
	Urine leaks when changes in body position or walking	27 (22.0)
	Frequent urine leaks	24 (19.5)
	Persistent urine leaks	9 (7.3)
	Persistent urine leaks without urination at all	9 (7.3)
Times of urine leaks	≤ once a week	43 (35.0)
	2 ~ 3 times a week	31 (25.2)
	Once a day	14 (11.4)
	Several times a day	22 (17.8)
	Always	13 (10.6)
The number of urinal pads changed by the patient (tablets/day)	0 ~ 1	54 (43.9)
	2 ~ 4	43 (35.0)
	5 ~ 7	11 (8.9)
	8 ~ 10	9 (7.3)
	> 10	6 (4.9)

**Table 5:** The score of three dimensions of patients with urinary incontinence compliance scale

Dimensions	Mean ± SD
pelvic floor muscle training compliance	2.76 ± 1.03
compliance of bladder training	2.94 ± 1.00
urination diary recording	3.20 ± 1.22
Total score	2.91 ± 0.95

**Table 6:** The items’ score of patients with urinary incontinence rehabilitation training compliance scale

Item	Mean ± SD
1. I can continue to contract my pelvic floor muscles for 2 to 10 s, relax, and rest for 2 to 10 s	2.62 ± 1.13
2. I can repeat the above actions 10 to 15 consecutive times, or for 5 to 15 min	2.80 ± 1.16
3. I can do the above actions (items 1 and 2) and train 3 to 8 times a day	2.81 ± 1.18
4. I can do pelvic floor exercises when standing, sitting, and lying down	2.84 ± 1.17
5. I can persist in pelvic floor muscle training for 3 months or more	2.75 ± 1.17
6. Before going to the toilet every time, I can contract my pelvic floor muscles until the sense of urgency disappears and then relax, and then urinate after 1 to 3 min	2.89 ± 1.17
7. I can gradually extend the interval between two urinations to 2 to 4 h as far as possible	2.86 ± 1.15
8. I can delay urination to make each urination more than 300 ml	3.03 ± 1.13
9. I can urinate regularly once every 2 h in the daytime and once every 4 h at night	3.04 ± 1.16
10. I can persist in bladder training for 2 months or more	2.92 ± 1.17
11. I can record a 24-h urination diary	3.25 ± 1.26
12. I can record urination diary for 3 to 7 days	3.16 ± 1.26

### Reliability

Cronbach’s α was 0.95 for the overall scale, and the Cronbach’s α values for the three factors were 0.93 of pelvic floor muscle training compliance, 0.91 of compliance of bladder training, and 0.94 of urination diary recording. The test–retest reliability was 0.82–0.87 for the items and 0.86 of the full scale (*P* < 0.05). The split-half reliability of this scale was 0.89 (*P* < 0.05).

#### Content validity

The S-CVI was 0.93 and the I-CVI was 0.87–1.0, indicating that the scale had good content validity (Table 7). The Spearman–Brown coefficient was 0.89, indicating that the items of the scale had high homogeneity and that the scale had good internal consistency. The CVR was 0.92, indicating that the experts recognised all of the items in the scale as essential. Kappa coefficient of the scale was 0.86 indicating that the items consistency in the scale were good [56].

**Table 7:** Kappa coefficient, CVI and CVR of items in the rehabilitation training compliance scale for patients with UI

Items	Kappa coefficient	CVI	CVR
Training of pelvic floor muscles			
1. I can continuously contract the pelvic floor muscles for 2–10 s, and relax for 2–10 s	0.86	0.95	1.00
2. I can repeat the movements for 10–15 times or 5–15 min continuously	0.86	1.00	0.81
3. I can repeat the movements for 3–8 times every day	0.86	0.95	1.00
4. I can exercise pelvic floor muscles when standing, sitting, and lying down	0.86	0.95	1.00
5. I can stick to pelvic floor exercises for 3 months or more	0.85	0.95	1.00
Bladder exercise			
6. Every time before going to toilet, I can contract pelvic floor muscles until the sense of urgency disappeared, after which I relax the muscles for 1–3 min before urination	0.86	0.91	1.00
7. I can gradually prolong the interval between two urinations to 2–4 h as possible	0.86	0.95	1.00
8. I can delay urination until the volume of each urination is > 300 mL	0.82	0.95	1.00
9. I am capable of timing urination, that is, once every 2 h in daytime and once every 4 h in night	0.87	0.95	1.00
10. I can stick to bladder exercise for 2 months or more	0.86	0.91	0.90
Urinary diary			
11. I can keep a diary of 24-h urination	0.85	0.87	0.90
12. I can stick to keeping urinary diary for 3–7 d	0.86	0.87	1.00
Scale for compliance evaluation	0.86	0.93	0.96

*CVI* Content Validity Index, *CVR* Content Validity Ratio

#### Construct validity

**Fig. 1:**
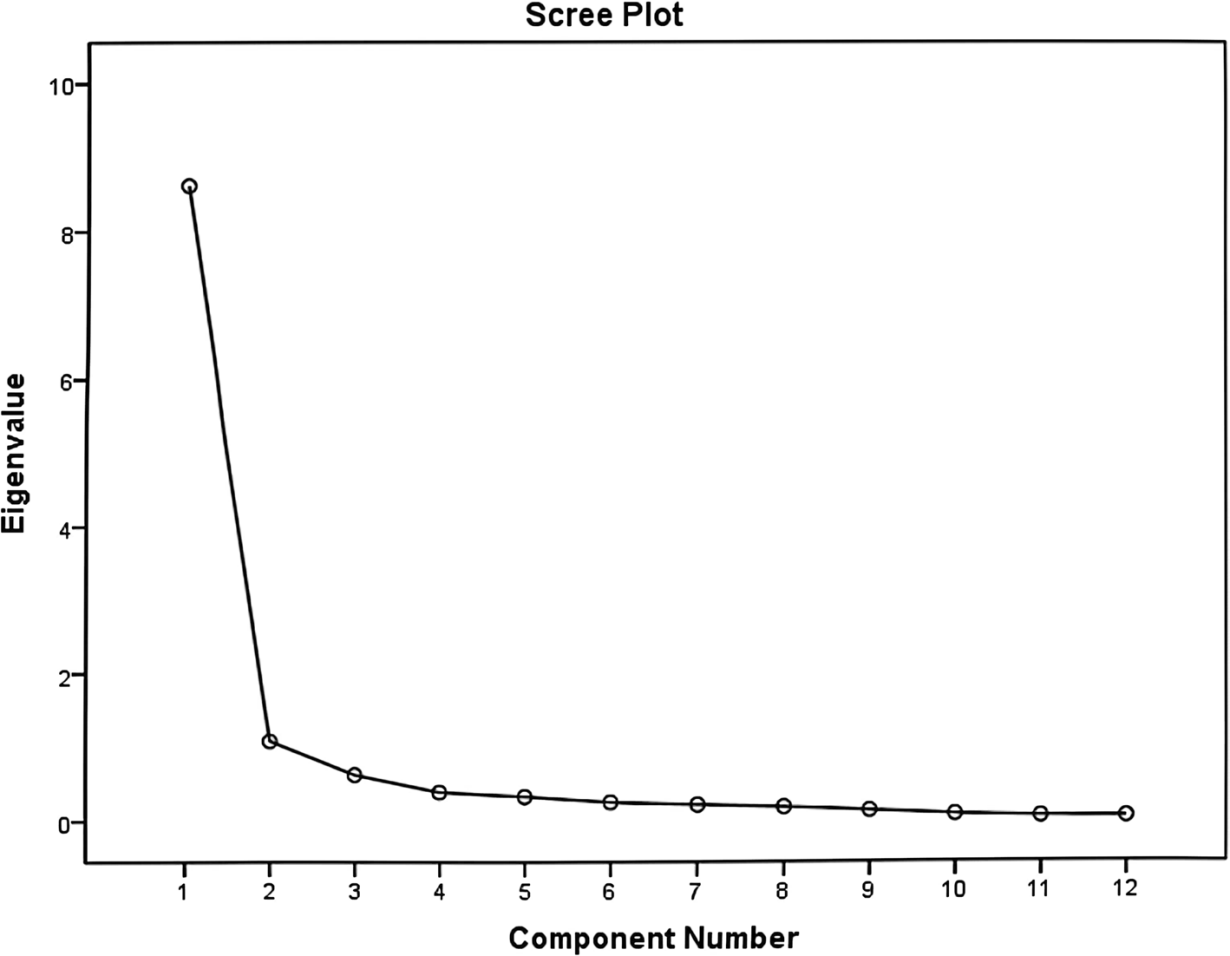
Scree plot

The KMO value was 0.90, indicating that factor analysis was appropriate for the data. Bartlett’s test of sphericity was significant (χ^2^ = 851.130, df = 66, *P* < 0.001), indicating that factor analysis was appropriate for the data. EFA with varimax rotation yielded a three-factor solution that explained 85.99% of the variance in the data (Table 8). The scree plot identified three factors that accounted for 85.99% of total variation in the data (Fig. 1). The common factors were generally in agreement with the hypothesised structures of the scale during design, indicating that the structure of the scale was appropriate. No items were loaded below 0.40, and no items were removed from the scale; hence, the scale was formed from 12 items. The details of the scale’s factor loading after varimax rotation with three factors are shown in Table 9. The three factors were designated ‘pelvic floor muscle training compliance’ (5 items), ‘compliance of bladder training (5 items)’, and ‘urination diary recording’ (2 items). Following the identification of a three-factor solution by EFA, CFA was performed to test the structure of the scale. Goodness-of-fit indices were examined to determine the degree of fit between the data and a hypothesised model. The goodness-of-fit indices were as follows: χ.^2^ = 134.964; df = 51; *p* < 0.001; CMIN/DF = 2.646; RMSEA = 0.116; CFI = 0.94; NFI = 0.91; TLI = 0.92; and IFI = 0.94 [49, 57].

**Table 8:** Factor analysis: total variance explained

Item	Initial eigenvalues			Extraction sums of squared loadings			Rotation sums of squared loadings		
	Total	Variance (%)	Accumulation (%)	Total	Variance (%)	Accumulation (%)	Total	Variance (%)	Accumulation (%)
1	8.620	71.837	71.837	8.620	71.837	71.837	3.982	33.186	33.186
2	1.076	8.963	80.799	1.076	8.963	80.799	3.924	32.703	65.889
3	.623	5.188	85.987	0.623	5.188	85.987	2.412	20.098	85.987
4	.389	3.239	89.226						
5	.329	2.740	91.966						
6	.249	2.077	94.043						
7	.215	1.793	95.836						
8	.184	1.535	97.372						
9	.134	1.120	98.492						
10	.080	.667	99.159						
11	.053	.438	99.597						
12	.048	.403	100.000

**Table 9:** Factor loading of the scale after varimax rotation with three factors

Item	Component 1	Component 2	Component 3
Item 1	0.516	0.614	0.286
Item 2	0.540	0.724	0.245
Item 3	0.474	0.801	0.137
Item 4	0.342	0.863	0.209
Item 5	0.23	0.847	0.287
Item 6	0.774	0.39	0.391
Item 7	0.771	0.391	0.302
Item 8	0.660	0.475	0.347
Item 9	0.757	0.287	0.349
Item 10	0.777	0.435	0.241
Item 11	0.307	0.261	0.899
Item 12	0.359	0.224	0.879

#### Convergent and discriminant validity

This scale showed good convergent and discriminant validity. The AVE and the square root of every AVE belonging to each factor were calculated, and the outcomes are shown in Table 10. There was a significant correlation between pelvic floor muscle training, bladder training, and urination diary recording (*p* < 0.05) in this scale, and the correlation coefficient was less than the corresponding AVE square root, indicating that the variables correlated with one another. There was also a certain degree of convergent and discriminant validity between the factors, and the scale had good convergent and discrimination validity.

**Table 10:** Convergent validity and discriminant validity

Factors	AVE	Factor 1	Factor 2	Factor 3
Factor 1	0.74	0.86		
Factor 2	0.70	0.83	0.84	
Factor 3	0.90	0.60	0.74	0.95

*AVE* Average variance extracted. On the diagonal, the square roots of every factor’s AVE were inserted to compare it with the other correlation coefficients

(*P* < 0.05)

#### Criterion-related validity

The criterion-related validity of the scale with the Chen PFMSE scale was 0.89 (*P* < 0.05), indicating that the scale had high calibration correlation validity.

## Discussion

This study developed a new scale to assess the training compliance of patients with UI, and its psychometric properties were assessed. The 12 items training compliance scale comprised three dimensions: ‘pelvic floor muscle training compliance,’ ‘compliance of bladder training,’ and ‘urination diary recording.’ The three dimensions performed well in reliability and content validity.

There were three steps in this scale development: the first step is a systematic literature review. To achieve the comprehensiveness of the literature review, it was completed under the guidance of a library staff who specialized in literature searching. The literature review developed the initial 25 items based on the characteristics of a rehabilitation training compliance scale for patients with UI. The second step was group discussions. To ensure the professionalism of the group discussion, the professional staff included in the group discussion were all related to the research area. After discussion, 12 items were extracted. The third step was Delphi consults. Twenty-two experts actively participated and gave constructive suggestions in the two rounds of Delphi consults. After the two rounds of Delphi consults, 8 items were revised. These three steps of scale development were rigorous and scientific, which ensured the objectivity, accuracy, effectiveness, and correlation of the scale items.

An effective response rate of 93.18% showed that the participants actively participated in the study and probably thought that this tool’s development would be helpful for their UI rehabilitation. The 12 items training compliance scale consisted of three factors. These three factors identified that the scale was meant to measure were extracted as was design. Even though the eigenvalue should be greater than one generally [58], clinical experts of the discussion group also suggested that the three factors should be retained in the scale. The accumulation of the extraction sums of the squared loadings and the scree plot also indicated that the three factors were reasonable. The CFA result suggested that the three-factor model fit the data. However, data analysis shows that the RMSEA is above 0.08, which indicates the model fit had a weakness.

The training compliance of patients with UI scale had good reliability, content validity, construct validity, convergent and discriminant validity, and criterion-related validity. This good reliability and validity indicate that this scale is a good measurement tool to assess the training compliance of patients with UI. This training compliance scale for patients with UI can facilitate the evaluation of training compliance, which can help medical staff examine patients’ weaknesses in training compliance and then develop specific interventions that improve the defective parts of training compliance for patients in the future.

The test–retest reliability and overall Cronbach’s α of this scale are as good as that of the Chen PFMSE scale. Compared with the Chen [26] PFMSE scale, this new scale’s I-CVI, S-CVI, the variance explained, and Criterion validity were better. The Chen PFMSE scale [26] were not reported the CFA, CVR, split-half reliability, Kappa coefficient, and convergent and discriminant validity, and these indexes performed well in the scale of training compliance of patients with UI. Compared with Chen’s scale development, this scale development process was more scientific and rigorous. Thus, the training compliance scale for patients with UI is a useful instrument for evaluating training compliance in patients with UI.

To our knowledge, there is no specific tool currently to assess UI patients' rehabilitation training compliance. This study constructed such an evaluation scale, using which medical staff can better know the rehabilitation training compliance of such patients. With the help of this scale, medical staff can promote rehabilitation training knowledge through online media, carry out health education lectures, interact with patients, provide personalised rehabilitation training guidance, and so on, all of which contribute to improving patients’ compliance with rehabilitation training [59]. Meanwhile, the scale can predict the recovery effectiveness and life quality of people with UI. Additionally, this scale could detect the weak aspects of training that people with UI do not comply with. This information could then be used to develop specific interventions to promote patient training compliance.

In summary, the development of the rehabilitation training compliance scale for patients with UI was scientific and strictly based on the scale designing principle. Therefore, this scale could be a reliable tool for medical staff to evaluate the rehabilitation training compliance of patients with UI.

This study has some limitations. First, the sample size of 123 patients with UI in this study was not big enough. Second, this study used convenience sampling; therefore, the representativeness of the sample could be insufficient. Third, this study unintentionally included some irrelevant information of the patients (for example, income). Fourth, items 1, 2, 3, 8, and 10 had cross-loadings (with two factors loading > 0.4). This overlap rate was too high, resulting in a slight weakness of the model’s fit. Therefore, the representativeness of the sample could be insufficient. Last, the minimum EFA and CFA sample affects the precision, stability, and replicability of the results. Affect by COVID-19, it was difficult to collect data at that time. Many people with UI were unable to seek medical treatment for UI is not an acute disease. Generally, for EFA and CFA, the stronger the data and the larger the sample, the more accurate the analysis will be. Further studies with larger sample sizes from a wider range of people are needed to validate the scale.

## Conclusion

This scale is a reliable, scientific tool to evaluate the compliance of patients with UI to rehabilitation training in clinical practices. The future study should be perfected the scale and use the scale to assess the compliance of patients. Further, explore the factors that affect the patient's compliance and formulate the intervention plan to improve the compliance according to the relevant factors to promote the patient's recovery and improve the patient's quality of life.

## Data Availability

All data generated or analysed during this study are included in this published article.

## References

[1] Tienza A, Robles JE, Hevia M, Algarra R, Diez-Caballero F, Pascual JI (2018). Prevalence analysis of urinary incontinence after radical prostatectomy and influential preoperative factors in a single institution. Aging Male.

[2] Radziminska A, Straczynska A, Weber-Rajek M, Styczynska H, Strojek K, Piekorz Z (2018). The impact of pelvic floor muscle training on the quality of life of women with urinary incontinence: a systematic literature review. Clin Interv Aging.

[3] Coyne KS, Kvasz M, Ireland AM, Milsom I, Kopp ZS, Chapple CR (2012). Urinary incontinence and its relationship to mental health and health-related quality of life in men and women in Sweden, the United Kingdom, and the United States. Eur Urol.

[4] Xue K, Palmer MH, Zhou F (2020). Prevalence and associated factors of urinary incontinence in women living in China: a literature review. BMC Urol.

[5] Burzynski B, Gibala P, Soltysiak-Gibala Z, Jurys T, Przymuszala P, Rzymski P, Stojko R (2022). How urinary incontinence affects sexual activity in polish women: results from a cross-sectional study. Int J Environ Res Public Health.

[6] Nambiar AK, Bosch R, Cruz F, Lemack GE, Thiruchelvam N, Tubaro A, Bedretdinova DA, Ambuhl D, Farag F, Lombardo R (2018). EAU guidelines on assessment and nonsurgical management of urinary incontinence. Eur Urol.

[7] Coyne KS, Wein A, Nicholson S, Kvasz M, Chen CI, Milsom I (2014). Economic burden of urgency urinary incontinence in the United States: a systematic review. J Manag Care Pharm.

[8] Todhunter-Brown A, Hazelton C, Campbell P, Elders A, Hagen S, McClurg D (2022). Conservative interventions for treating urinary incontinence in women: an overview of cochrane systematic reviews. Cochrane Database Syst Rev.

[9] Hu JS, Pierre EF (2019). Urinary incontinence in women: evaluation and management. Am Fam Physician.

[10] Hagen S, Bugge C, Dean SG, Elders A, Hay-Smith J, Kilonzo M, McClurg D, Abdel-Fattah M, Agur W, Andreis F (2020). Basic versus biofeedback-mediated intensive pelvic floor muscle training for women with urinary incontinence: the OPAL RCT. Health Technol Assess.

[11] Kaya S, Akbayrak T, Gursen C, Beksac S (2015). Short-term effect of adding pelvic floor muscle training to bladder training for female urinary incontinence: a randomized controlled trial. Int Urogynecol J.

[12] Kegel AH (1948). Progressive resistance exercise in the functional restoration of the perineal muscles. Am J Obstet Gynecol.

[13] Qaseem A, Dallas P, Forciea MA, Starkey M, Denberg TD, Shekelle P (2014). Clinical guidelines committee of the American college of P: nonsurgical management of urinary incontinence in women: a clinical practice guideline from the American college of physicians. Ann Intern Med.

[14] Colombo R, Pellucchi F, Moschini M, Gallina A, Bertini R, Salonia A, Rigatti P, Montorsi F (2015). Fifteen-year single-centre experience with three different surgical procedures of nerve-sparing cystectomy in selected organ-confined bladder cancer patients. World J Urol.

[15] Kretschmer A, Grimm T, Buchner A, Grimm J, Grabbert M, Jokisch F, Schneevoigt BS, Apfelbeck M, Schulz G, Bauer RM (2017). Prognostic features for objectively defined urinary continence after radical cystectomy and ileal orthotopic neobladder in a contemporary cohort. J Urol.

[16] Soave I, Scarani S, Mallozzi M, Nobili F, Marci R, Caserta D (2019). Pelvic floor muscle training for prevention and treatment of urinary incontinence during pregnancy and after childbirth and its effect on urinary system and supportive structures assessed by objective measurement techniques. Arch Gynecol Obstet.

[17] Cacciari LP, Dumoulin C, Hay-Smith EJ (2019). Pelvic floor muscle training versus no treatment, or inactive control treatments, for urinary incontinence in women: a cochrane systematic review abridged republication. Braz J Phys Ther.

[18] Sawettikamporn W, Sarit-Apirak S, Manonai J (2022). Attitudes and barriers to pelvic floor muscle exercises of women with stress urinary incontinence. BMC Womens Health.

[19] Lahdenpera TS, Wright CC, Kyngas HA (2003). Development of a scale to assess the compliance of hypertensive patients. Int J Nurs Stud.

[20] Venegas M, Carrasco B, Casas-Cordero R (2018). Factors influencing long-term adherence to pelvic floor exercises in women with urinary incontinence. Neurourol Urodyn.

[21] Woodley SJ, Lawrenson P, Boyle R, Cody JD, Morkved S, Kernohan A, Hay-Smith EJC (2020). Pelvic floor muscle training for preventing and treating urinary and faecal incontinence in antenatal and postnatal women. Cochrane Database Syst Rev.

[22] Sheng Y, Carpenter JS, Ashton-Miller JA, Miller JM (2022). Mechanisms of pelvic floor muscle training for managing urinary incontinence in women: a scoping review. BMC Womens Health.

[23] Wein AJ (2002). Pelvic floor muscle training for urinary incontinence in women. J Urol.

[24] Widdison R, Rashidi A, Whitehead L (2022). Effectiveness of mobile apps to improve urinary incontinence: a systematic review of randomised controlled trials. Bmc Nurs.

[25] Borello-France D, Burgio KL, Goode PS, Ye W, Weidner AC, Lukacz ES, Jelovsek JE, Bradley CS, Schaffer J, Hsu Y (2013). Adherence to behavioral interventions for stress incontinence: rates, barriers, and predictors. Phys Ther.

[26] Chen SY (2004). The development and testing of the pelvic floor muscle exercise self-efficacy scale. J Nurs Res.

[27] Sacomori C, Cardoso FL, Porto IP, Negri NB (2013). The development and psychometric evaluation of a self-efficacy scale for practicing pelvic floor exercises. Braz J Phys Ther.

[28] Porta Roda O, Diaz Lopez MA, VaraPaniagua J, Simo Gonzalez M, Diaz Bellido P, Espinos Gomez JJ (2016). Adherence to pelvic floor muscle training with or without vaginal spheres in women with urinary incontinence: a secondary analysis from a randomized trial. Int Urogynecol J.

[29] Palmer SJ (2020). Urge incontinence in postmenopausal women. Br J Community Nurs.

[30] Aydın Sayılan A, Özbaş A (2018). The effect of pelvic floor muscle training on incontinence problems after radical prostatectomy. Am J Mens Health.

[31] Tostivint V, Verhoest G, Cabarrou B, Gas J, Coloby P, Zgheib J, Thoulouzan M, Soulie M, Game X, Beauval JB (2021). Quality of life and functional outcomes after radical cystectomy with ileal orthotopic neobladder replacement for bladder cancer: a multicentre observational study. World J Urol.

[32] Sigurdardottir T, Steingrimsdottir T, Geirsson RT, Halldorsson TI, Aspelund T, Bø K (2020). Can postpartum pelvic floor muscle training reduce urinary and anal incontinence?: An assessor-blinded randomized controlled trial. Am J Obstet Gynecol.

[33] Dumoulin C, Alewijnse D, Bo K, Hagen S, Stark D, Van Kampen M, Herbert J, Hay-Smith J, Frawley H, McClurg D (2015). Pelvic-floor-muscle training adherence: tools, measurements and strategies-2011 ICS state-of-the-science seminar research paper II of IV. Neurourol Urodyn.

[34] Newman DK, Borello-France D, Sung VW (2018). Structured behavioral treatment research protocol for women with mixed urinary incontinence and overactive bladder symptoms. Neurourol Urodyn.

[35] Wallace SA, Roe B, Williams K, Palmer M (2004). Bladder training for urinary incontinence in adults. Cochrane Database Syst Rev.

[36] Yildiz N. “Intravaginal eletrical stimulation for bladder training method” by Cassio L.Z. Riccetto, 2021. Int Braz J Urol. 2022;48(2):373–4. 10.1590/S1677-5538.IBJU.2021.0817 PMC8932040 35170905

[37] Firinci S, Yildiz N, Alkan H, Aybek Z (2020). Which combination is most effective in women with idiopathic overactive bladder, including bladder training, biofeedback, and electrical stimulation? A prospective randomized controlled trial. Neurourol Urodyn.

[38] Khandelwal C, Kistler C (2013). Diagnosis of urinary incontinence. Am Fam Physician.

[39] Pauls RN, Hanson E, Crisp CC (2015). Voiding diaries: adherence in the clinical setting. Int Urogynecol J.

[40] Vaccari NA, da Silveira LTY, Bortolini MAT, Haddad JM, Baracat EC, Ferreira EAG (2020). Content and functionality features of voiding diary applications for mobile devices in Brazil: a descriptive analysis. Int Urogynecol J.

[41] Tsang HW, Fung KM, Corrigan PW (2006). Psychosocial treatment compliance scale for people with psychotic disorders. Aust N Z J Psychiatry.

[42] Kimberlin CL, Winterstein AG (2008). Validity and reliability of measurement instruments used in research. Am J Health-Syst Ph.

[43] Weir JP (2005). Quantifying test-retest reliability using the intraclass correlation coefficient and the SEM. J Strength Cond Res.

[44] Rattray J, Jones MC (2007). Essential elements of questionnaire design and development. J Clin Nurs.

[45] Atashzadeh-Shoorideh F, Parvizy S, Hosseini M, Raziani Y, Mohammadipour F (2022). Developing and validating the nursing presence scale for hospitalized patients. Bmc Nurs.

[46] Subali B (2018). Content validity analysis of first semester formative test on biology subject for senior high school. J Phys.

[47] Ayre C, Scaly AJ (2014). Critical values for Lawshe’s content validity ratio. Meas Eval Couns Dev.

[48] Kyriazos TA (2018). Applied psychometrics: sample size and sample power Considerations in Factor Analysis (EFA, CFA) and SEM in general. Psychology.

[49] Kline RB (1998). Software review: Software programs for structural equation modeling: Amos, EQS, and LISREL. J Psychoeduc Assess.

[50] Chen X, Yu Q, Yu F, Huang Y, Zhang L. Psychometric evaluation of the Chinese version of the Snizek-revised hall’s professionalism inventory scale. J Int Med Res. 2019;47(3):1154–68. 10.1177/0300060518817401 PMC6421374 30614338

[51] Bick D, Bishop J, Coleman T, Dean S, Edwards E, Frawley H, Gkini E, Hay-Smith J, Hemming K, Jones E (2022). Antenatal preventative pelvic floor muscle exercise intervention led by midwives to reduce postnatal urinary incontinence (APPEAL): protocol for a feasibility and pilot cluster randomised controlled trial. Pilot Feasibil Stud.

[52] Hagen S, Elders A, Stratton S, Sergenson N, Bugge C, Dean S, Hay-Smith J, Kilonzo M, Dimitrova M, Abdel-Fattah M (2020). Effectiveness of pelvic floor muscle training with and without electromyographic biofeedback for urinary incontinence in women: multicentre randomised controlled trial. BMJ.

[53] Chen X, Luo L, Jiang L, Shi L, Yang L, Zeng Y, Li F, Li L. Development of the nurse’s communication ability with angry patients scale and evaluation of its psychometric properties. J Adv Nurs. 2021;77(6):2700–8. 10.1111/jan.14788 PMC8248006 33629754

[54] Huber CA, Reich O (2016). Medication adherence in patients with diabetes mellitus: does physician drug dispensing enhance quality of care? Evidence from a large health claims database in Switzerland. Patient Prefer Adherence.

[55] de Raadt A, Warrens MJ, Bosker RJ, Kiers HAL (2021). A Comparison of reliability coefficients for ordinal rating scales. J Classif.

[56] Qiang L (2021). Reliability and validity analysis of the revised SERVQUAL scale on the quality evaluation of community hypertension health management. China Health Stat.

[57] Browne MW, Cudeck R. Alternative Ways of Assessing Model Fit. Sociological Methods & Research. 1992;21(2):230–58.

[58] Hair JF, Black WC, Babin BJ, Anderson RE, Tatham RL. Multivariate Data Analysis. 7th Ed. Upper Saddle River: Prentice Hall; 2010.

[59] Ostaszkiewicz J, Tomlinson E, Hunter K. The effects of education about urinary incontinence on nurses’ and nursing assistants’ knowledge, attitudes, continence care practices, and patient outcomes: a systematic review. J Wound Ostomy Continence Nurs. 2020;47(4):365–80. 10.1097/WON.0000000000000651 33290014

